# Evaluating four major algorithms for identifying differential regulators in condition-specific transcriptional responses

**DOI:** 10.1186/1471-2105-15-S10-P33

**Published:** 2014-09-29

**Authors:** Hui Yu, Zhongming Zhao

**Affiliations:** 1 Department of Biomedical Informatics, Vanderbilt University School of Medicine, Nashville, TN 37232, USA; 2 Department of Cancer Biology, Vanderbilt University School of Medicine, Nashville, TN 37232, USA; 3 Center for Quantitative Sciences, Vanderbilt University Medical Center, Nashville, TN 37232, USA

## Background

Identifying molecular regulators underlying condition-specific transcriptional responses is essential for our understanding of their underlying molecular mechanisms. So far, there have been several computational methods developed for this purpose. Specifically, four major algorithms, TFact [1], RIF [2], CSA [3], and DRrank [4], were released one after another. Each of these algorithms has its own features and evaluation strategies. Thus, these methods should be systematically evaluated so that the users can make the most appropriate method selection for their practical application needs.

## Materials and methods

In this work, we evaluated the four algorithms using *Escherichia coli* transcription network models and synthetic expression datasets that were generated by SynTReN [5] and GeneNetWeaver [6]. Specifically, we developed a simulation-based schema to evaluate each algorithm according to operatively defined, known “differential regulators.” In addition, we tested each method’s robustness against its key parameter(s) and explored factors that influence algorithm performance in general.

## Results

We found that TFact and DRrank stood out as the best methods in terms of both accuracy and robustness. In total, there were seven cases in which one single regulator was artificially perturbed. TFact attained the closest approximation four times, while DRrank had the best performance the other three times. In another 15 scenarios in which there were perturbations of multiple regulators, DRrank was ranked on average the best algorithm, while TFact had the second-place rank. Based on these observations, TFact and DRrank may each be best applied to different circumstances: TFact is better used for single regulator implication, while DRrank is better for multiple regulators’ simultaneous perturbation. In general, we observed that algorithms’ performances were negatively correlated with the number of regulation links per target, which may indicate that independent regulators are easier to recover than synergistic ones.

## Conclusions

This work represents a preliminary benchmarking evaluation of four major currently-available algorithms for differential regulator identification. According to our simulation-based evaluation paradigm, two of the published algorithms, TFact and DRrank, are more robust than the other two. Further evaluation is needed, such as applying them to specific human disease expression datasets under different conditions or different platforms. Furthermore, these algorithms may be extended to rapidly emerging next-generation sequencing (NGS) data.
